# Boredom proneness and Chinese vocational college students academic disengagement: mediating role of academic self-efficacy and moderating role of self-control

**DOI:** 10.3389/fpsyg.2025.1687667

**Published:** 2026-01-13

**Authors:** Yantao Shi, Xueli Hui, Guanghai Li, Mingkun Ouyang, Cheng Yang

**Affiliations:** 1 Faculty of Education, Guangxi Normal University, Guilin, China; 2 College of Innovation and Entrepreneurship, Guangxi Minzu University, Nanning, China; 3 College of Preschool Education, Chongqing Youth Vocational & Technical College, Chongqing, China; 4 School of Educational Sciences, Guangxi Minzu University, Nanning, China; 5 School of Foreign Studies, Guangxi Minzu University, Nanning, China

**Keywords:** academic disengagement, academic self-efficacy, boredom proneness, self-control, vocational college students

## Abstract

**Objective:**

This study aimed to explore the relationship between boredom proneness and academic disengagement among vocational college students, specifically examining the mediating role of academic self-efficacy and the moderating effect of self-control in this relationship.

**Design:**

The study employed a cross-sectional design to investigate the direct and indirect pathways between boredom proneness and academic disengagement. It also explored whether self-control would moderate these relationships.

**Method:**

The participants were 2,687 vocational college students from Guangxi, China, who were recruited through a convenience sampling method. The students completed measures assessing boredom proneness, academic disengagement, academic self-efficacy, and self-control. Confirmatory factor analysis (CFA), Pearson correlation analyses, and PROCESS macro analyses were used to examine measurement validity and test the mediation and moderated mediation models.

**Results:**

Boredom proneness positively predicted academic disengagement. Academic self-efficacy partially mediated this association. Self-control moderated the path from boredom proneness to academic self-efficacy and the path from academic self-efficacy to academic disengagement. The indirect effect of boredom proneness on academic disengagement through academic self-efficacy was stronger under high selfcontrol, whereas it was weaker and non-significant under low selfcontrol.

**Conclusion:**

Self-control should not be interpreted as a simple buffering factor in this model. Interventions should combine academic self-efficacy support with self-regulatory support.

## 1 Introduction

Academic disengagement is one of the most severe issues in education today (Lin, 2017). It has been defined in relevant research as “a lack of effort or persistence” (Skinner et al., 2009). However, academic disengagement is not only about “lack of effort,” but also includes withdrawal behaviors (Skinner et al., 2009; Wang et al., 2017), truancy, and even leads to school dropout (Bergdahl et al., 2020; Tafelski et al., 2017). Previous studies have found that academic disengagement encompasses four dimensions: behavioral, emotional, cognitive, and volitional disengagement (Xue, 2023). Behavioral disengagement refers to being easily distracted, displaying perfunctory attitudes, and quickly abandoning efforts in the classroom (Jang et al., 2016; Xue, 2023). Emotional disengagement involves experiencing negative emotions and energy depletion during learning tasks, such as sadness, stress, or emotional conflict (Xue, 2023). Cognitive disengagement refers to using disorganized, unplanned, and unstructured strategies when completing tasks (Xue, 2023). And volitional disengagement refers to students passively accepting teaching without expressing needs, participating in discussions, or attempting to align learning content with personal interests, goals, or preferences (Xue, 2023; Reeve, 2013). Research has also found that students displaying academic disengagement tend to be disruptive, unwilling to set high academic goals, have lower grades, and face higher risks of dropout. And they are more passive, struggling to cope with emotions such as boredom, anxiety, and even anger (Reyes et al., 2012). Furthermore, academic disengagement negatively affects students’ self-perception and academic self-efficacy. Negative cognition tend to lead to overgeneralization and trigger “learned helplessness” (Klein et al., 1976), resulting in difficulties in coping with challenges in other areas (such as extracurricular activities or interpersonal relationships). These students typically underestimate their abilities and lack motivation, thus becoming trapped in the vicious cycle of “anticipated failure—avoidance behavior—outcome confirmation.” Low self-efficacy further exacerbates their maladaptive behavior patterns.

Boredom is defined as “the aversive experience of wanting to engage in satisfying activities but being unable to do so” (Eastwood et al., 2012, p. 482; Yang et al., 2020). Boredom proneness refers to an individual’s stable tendency to experience boredom across various situations (Fahlman et al., 2013). The stimuli associated with boredom proneness include repetition, lack of novelty, and monotony (Pawlak et al., 2020; Kruk et al., 2021; Perkins and Hill, 1985). Boredom proneness is commonly linked to social and psychological issues (Lepera, 2011; Dursun, 2016; Elpidorou, 2014). Todman et al. (2008) suggests that persistent boredom proneness may increase risk-taking behaviors and the pursuit of material rewards, disrupt positive emotions, lead to distraction, reduce cognitive efficiency, and result in a generally heightened lack of interest. Previous research indicates that boredom proneness can trigger a range of adverse psychosocial outcomes, including negative emotions such as depression and anxiety (Lepera, 2011), cognitive errors (Wallace et al., 2003), poor academic performance (Tze et al., 2016), and problem behaviors such as substance abuse (Weybright et al., 2015). Additionally, boredom proneness can provoke individuals to seek external stimuli and tend to pursue short-term sensory rewards, potentially leading to dependency on short-term pleasure and resulting in maladaptive behaviors such as internet addiction and substance abuse. Furthermore, studies have shown that boredom proneness may also affect an individual’s self-regulation abilities, such as difficulties in emotion regulation and heightened impulsivity (Baumeister et al., 2007). Chronic boredom may cause individuals to lose interest in external activities, generate negative emotions, and lead to social alienation and emotional isolation (Eastwood et al., 2012; Tagliaferri et al., 2025), thereby severely impairing an individual’s social adaptability and interpersonal interactions. Protracted feelings of boredom may cause individuals to lose interest in external activities, engender negative emotions, trigger social disengagement and emotional isolation (Eastwood et al., 2012; Tagliaferri et al., 2025), thereby severely impairing their capacity for social adaptation and interpersonal interaction.

### 1.1 Boredom proneness and academic disengagement

Boredom proneness is positively associated with academic disengagement. According to the Attentional Resource Theory, individuals’ attention is limited and easily distracted (Basil, 1994). When students experience boredom, their attention may be diverted, leading them to disengage from learning tasks (Cvetković and Opsenica-Kostić, 2024). This distraction of attention is closely related to students’ learning outcomes. Students with high boredom proneness often struggle to concentrate, resulting in poor learning performance and subsequent academic disengagement (Solhi, 2021). Research indicates that students with strong boredom proneness are more likely to experience fatigue and a sense of powerlessness, leading to academic burnout (Aeni et al., 2023). Intense boredom proneness causes students to lack positive emotions when facing academic challenges (Adesola et al., 2019), and learning tasks gradually become burdensome and tedious, ultimately contributing to academic disengagement. Further studies have shown that students who feel bored are more likely to exhibit emotional exhaustion and lack of motivation (Zhao and Wang, 2025; Liu and Xie, 2024), which can eventually lead to long-term academic withdrawal and dropout. Boredom proneness is influenced not only by students’ individual psychological characteristics and attention, but also by cultural and educational system factors. Related studies show that cultural factors such as “promotion is king,” “academic qualifications are paramount,” weak social identity for vocational education and other cultural factors lead to study-weary behavior (Zeng et al., 2024; Zhang et al., 2025). For example, competitive values of Confucian culture may lead students to pay too much attention to academic performance, resulting in higher academic pressure, which in turn affects their learning attitudes and behaviors (Chen, 2025; Hau and Ho, 2010). In addition, China’s education is becoming more and more convoluted, causing students to neglect personal interests and learning autonomy in pursuit of surpassing their peers, aggravating students’ academic burden and anxiety (Yang et al., 2020; Li et al., 2021), which leads students to easily fall into boredom and burnout in learning, exacerbating the risk of academic disengagement (Chen and Leung, 2024; Ding and Si, 2024).

**Figure 1:**
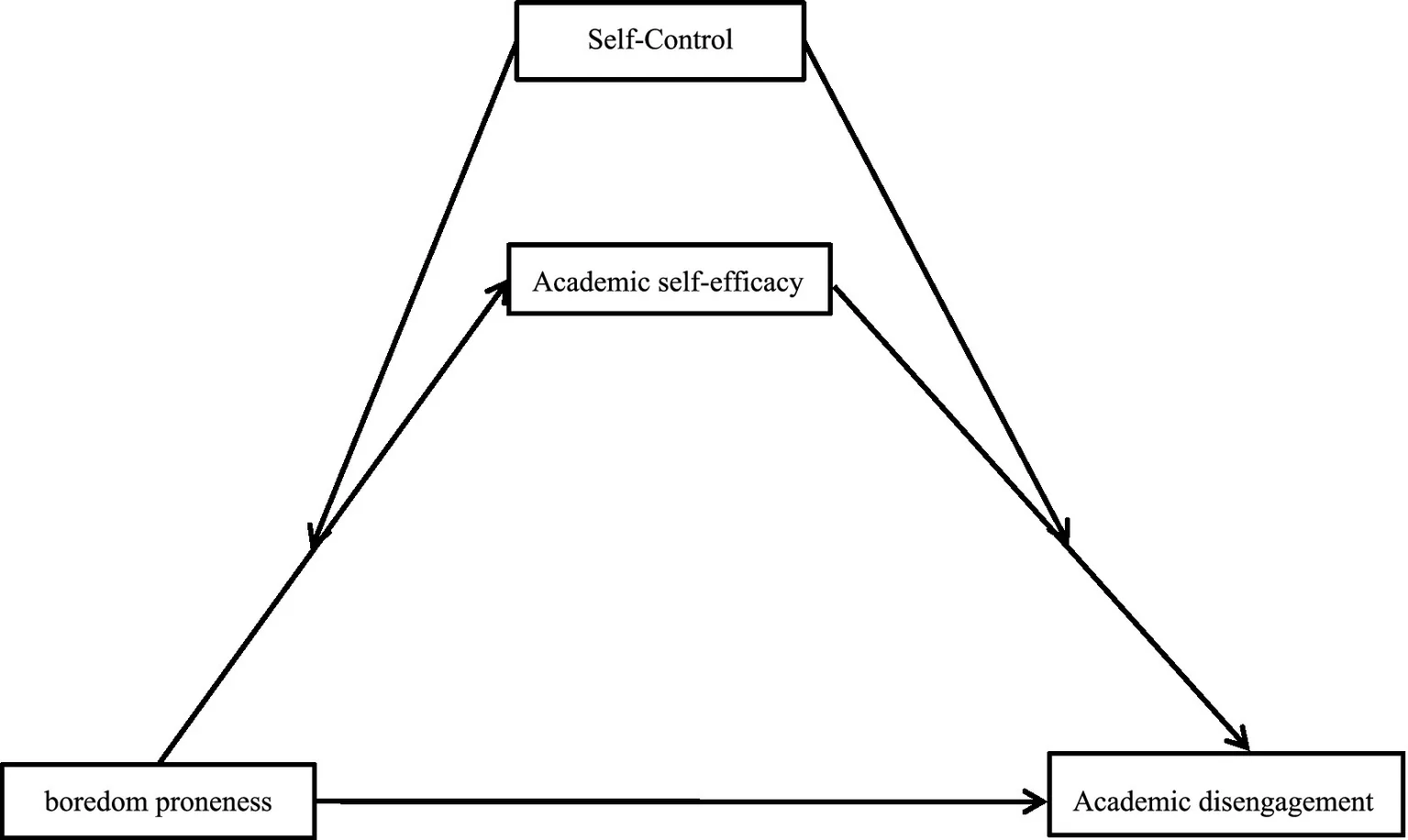
The assumed moderated mediation model.

Boredom proneness in students permeates various aspects of their learning activities and academic development. Students’ ability to perceive and manage boredom has significant effects on their physical and mental development (Mikulas and Vodanovich, 1993; Kula, 2022), as well as on their subsequent learning attitudes and behaviors. Recent studies on students’ boredom proneness have revealed its impact on students’ learning emotions and behaviors. For instance, meta-analyses have found that boredom proneness is negatively correlated with emotional regulation ability and academic self-efficacy (Affandi et al., 2025; Tze et al., 2016), while positively correlated with academic burnout (Li et al., 2025). These studies indirectly suggest that boredom proneness is an important predictor of academic disengagement. Kula (2022) and Deveci (2016) confirmed the close relationship between the two, and further highlighted a significant positive correlation between boredom proneness and academic disengagement. These studies contribute to understanding the direct relationship between boredom proneness and academic disengagement. However, the internal mechanisms (such as mediating and moderating mechanisms) through which boredom proneness influences academic disengagement remain unclear and require further investigation. To address the issue, this study aims to construct a moderated mediation model that examines the mediating role of academic self-efficacy in the relationship between boredom proneness and academic disengagement, as well as the moderating role of self-control within this framework. This model holds theoretical significance, it helps address questions such as how boredom proneness affects academic disengagement, and under what conditions this influence becomes more pronounced (see Figure 1).

### 1.2 Mediating role of academic self-efficacy

Academic self-efficacy refers to an individual’s belief in their ability to learn and successfully complete academic tasks at a specified level (Bandura, 1997; Schunk and DiBenedetto, 2020; Yi et al., 2025). In a state of boredom, students may experience negative emotions and physiological arousal (such as anxiety, fatigue, etc.), which can interfere with their learning and task performance. According to the self-efficacy theory, students’ ability to regulate emotions and arousal directly impacts their academic self-efficacy and performance in academic tasks (Bandura, 1997). If students are unable to effectively regulate negative arousal, it may lead to a decline in their academic self-efficacy (Medrano et al., 2016). Willis (2014) and Van Hooft and Van Hooff (2018) further point out that boredom proneness, as a negative emotional experience, implies that individuals may struggle to regulate the relationship between internal emotional responses and external behaviors when facing learning tasks. Therefore, boredom proneness may negatively impact subsequent academic self-efficacy.

Empirical studies have supported the relationship between boredom proneness and students’ academic self-efficacy. Bench and Lench (2019) found that boredom proneness, as an emotional experience, significantly affects an individual’s emotional regulation ability, i.e., boredom proneness can significantly predict self-efficacy. In addition, Liu and Lu (2014) conducted a study on the relationship between boredom proneness and academic self-efficacy, providing more direct empirical evidence for the connection between the two. They found a negative correlation between boredom proneness and academic self-efficacy. Based on the above theoretical and empirical research, this study proposes *Hypothesis 1a*: Boredom proneness is significantly negatively correlated with academic self-efficacy.

Academic self-efficacy is significantly negatively correlated with students’ academic disengagement. According to the Job Demands-Resources (JD-R) model, positive psychological resources such as self-efficacy, emotional intelligence, optimism, and motivation are considered important personal resources that can enhance learning engagement and motivation. These resources help individuals better cope with academic challenges, thereby reducing academic burnout (Barr, 2014; Providas, 2016; Guglielmi et al., 2012; Xanthopoulou et al., 2007). Meta-analytic results indicate a significant positive correlation between self-efficacy and learning engagement and motivation (Chang and Chien, 2015; Fatimah et al., 2024). Students with higher levels of learning engagement and motivation have a greater sense of identification with the knowledge and skills they learn, and are more willing to invest additional effort, thereby reducing academic burnout (Reyes-De-Cózar et al., 2023; Cazan, 2015). Based on the theoretical analysis above, we hypothesize that academic self-efficacy is significantly negatively correlated with students’ academic disengagement.

Existing empirical studies support this hypothesis, although most research has primarily focused on the relationship between academic self-efficacy and academic disengagement at the secondary school level. For instance, some studies have found a significant positive correlation between students’ academic self-efficacy and learning motivation (Kim, 2013). Additionally, students with higher academic self-efficacy tend to show lower levels of academic burnout and higher academic engagement (Liu, 2025; Anshorryyah and Hadinata, 2023). However, these studies predominantly focus on the secondary school stage, with limited exploration of the role of academic self-efficacy in vocational college students. How academic self-efficacy interacts with academic disengagement in vocational college students still requires further investigation. Therefore, this study proposes *Hypothesis 1b*: There is a significant negative correlation between academic self-efficacy and academic disengagement in vocational college students.

Based on the significant correlation between boredom proneness and academic self-efficacy, as well as the significant negative correlation between academic self-efficacy and academic disengagement, this study proposes that academic self-efficacy may play a significant mediating role between boredom proneness and academic disengagement. Although existing research has not directly tested this mediating effect, indirect evidence supports this hypothesis. For example, studies have found that academic self-efficacy mediates the relationship between academic burnout and its predictors (e.g., academic motivation, emotional regulation ability) in secondary school students (Supervía and Quílez Robres, 2021).

### 1.3 Moderating role of self-control

First, this study proposes the role of self-control in the relationship between boredom proneness and academic efficacy. Self-control refers to the ability of an individual to resist and suppress internal desires and external temptations that may interfere with the pursuit of long-term goals (Tangney et al., 2004). According to the theory of emotion regulation, self-control is central to regulating emotions, especially when dealing with strong negative emotions such as boredom (Gross, 2002; Baumeister and Vohs, 2004). As a common negative emotion, boredom is usually accompanied by depression, lack of motivation and cognitive stagnation (Grazia et al., 2021; van Hooft and van Hooff, 2018). Research indicates that students with elevated boredom proneness typically exhibit diminished self-control, experience more frequent negative affect, and struggle to deploy effective coping strategies when confronted with academic demands—ultimately eroding their sense of academic efficacy (Amiri et al., 2022; Hubbard, 2019; Finkielsztein, 2019). Individuals with high self-control can effectively manage boredom through emotion regulation mechanism, adopt strategies to alleviate boredom, enhance learning engagement and persistence, and maintain high academic efficacy (Grazia et al., 2021). Relatively speaking, low self-control individuals are difficult to regulate emotions and prone to avoidance behavior, resulting in decreased academic efficacy. Studies have also shown that self-control can effectively mitigate the negative impact of boredom on academic efficacy (Mugon et al., 2020; Bieleke et al., 2020). Based on the above conclusions, we speculate that self-control can be regarded as an important moderating variable to reduce the negative impact of boredom proneness on academic efficacy.

Secondly, self-control is proposed as a moderator of the relationship between academic self-efficacy and academic disengagement. According to cognitive-behavioral theory (CBT), self-control as a core mental ability is essential for the regulation of emotion and behavior (Kruschwitz et al., 2024; Thorpe and Olson, 1990). It helps individuals regulate negative emotions and reduces the interference of emotional dissonance with cognitive processing, thereby reducing the negative impact of negative emotions on academic task performance (Curtis, 2009; Stiller, 2020). Academic efficacy is closely related to emotional and cognitive assessment (Chen and Leung, 2024; Putwain et al., 2013). Negative cognition (e.g., low self-efficacy) often leads to negative emotions (such as anxiety and depression), which further aggravate academic avoidance and academic burnout. We hypothesized that students with high self-control could improve their assessment of academic tasks, maintain positive emotions, enhance academic efficacy, and effectively cope with academic challenges through emotional regulation and cognitive restructuring. These students can rationally assess task difficulty, set feasible goals, and maintain learning motivation through emotional regulation, and finally achieve higher learning performance. These regulatory abilities provide psychological resources for coping with academic stress and help prevent academic burnout and academic disengagement. This view has also been indirectly supported by studies showing that students with high self-control are better able to manage negative emotions, avoid distractions caused by emotional exhaustion, and thus reduce academic disengagement (King and Gaerlan, 2014; Tripathi et al., 2023; Goetz et al., 2015). In addition, Allahverdipour et al. (2006) found that self-control was negatively correlated with academic disengagement, indicating that students with higher self-control are more effective at coping with academic stress, sustaining positive attitudes, and consequently reducing academic disengagement. To sum up, self-control as a moderating variable affects the relationship between academic efficacy and academic disengagement.

### 1.4 Purpose of the study

In summary, this study explores the mediating role of academic self-efficacy in the relationship between boredom proneness and academic disengagement among vocational college students, as well as the moderating role of self-control in the direct and indirect relationships between boredom proneness and academic disengagement. This study aims to answer the psychological mechanisms underlying the close connection between boredom proneness and academic disengagement among vocational college students under the influence of self-control.

## 2 Methods

### 2.1 Participants

Two thousand six hundred eighty-seven Vocational College Students recruited from China. Using a convenient sample, students were invited to complete a questionnaire through the Questionnaire Star (ww.wjx.cn). The sample included 1986 females (73.9%) and males 701 (26.1%). The average age was 19.28 (*SD* = 1.21), ranging from 16 to 29. With regard to their geographical origin, 347 (12.9%) were from cities, a total of 2,340 (87.1%) originate from rural regions. With respect to their grade level in academia, 37.4% are currently in their first year of vocational college, 47.3% are currently in their second year of vocational college, and 15.2% are currently in their third year of vocational college.

### 2.2 Measures

#### 2.2.1 Boredom proneness

The Boredom Proneness Scale (BPS; Farmer and Sundberg, 1986) was used to assess the level of boredom proneness among vocational college students The scale includes 12 items in 2 dimensions: 6 items about internal (e.g., “It is easy for me to concentrate on my activities.”), 6 items about external (e.g., “When I was young, I was often in monotonous and tiresome situations.”). Each item was rated on a 7-point Likert scale ranging from 1 (*totally matched*) to 7 (*totally mismatched*) with higher average scores indicating higher levels of boredom proneness. The second-order CFA model showed that the ELS had good construct validity, with *χ*^2^/df = 10.748, RMSEA = 0.060, NFI = 0.972, GFI = 0.974, TLI = 0.961, CFI = 0.975. In this study, the overall scale’s internal consistency reliability coefficient is 0.844.

#### 2.2.2 Academic disengagement

The academic disengagement scale (ADS) developed by Chen (2025) was used to measure vocational college students’ academic disengagement scales. The scale consists of 21 items in 2 dimensions: behavioral disengagement (11 items, e.g., “I devote very little time and energy to studying.”), emotional disengagement (10 items, e.g., I feel very tired when I have to face studying when I wake up in the morning). Each item was rated on a 5-point Likert scale from *1* (*completely inconsistent*) to *5* (*completely consistent*), and higher average scores indicated higher levels of academic disengagement. The second-order CFA model showed that the OSI had good construct validity, with *χ*^2^/df = 7.484, RMSEA = 0.049, NFI = 0.976, GFI = 0.977, TLI = 0.968, CFI = 0.979. In this study, Cronbach’s alpha was 0.894.

#### 2.2.3 Academic self-efficacy

The academic self-efficacy was measured using the Chinese version of the Academic self-efficacy (AS) (Pintrich and DeGroot, 1990), revised by Liang and Zhou (2000) to fit the purpose of the study on Chinese students. TheThe scale consists of 11 items (e.g., “When thinking about a problem, I can connect the knowledge I have learned before and after.”). Each item was rated on a 5-point Likert scale from ranging from *1* (*completely inconsistent*) to *5* (*completely consistent*) with higher average scores indicating higher levels of academic self-efficacy. The second-order CFA model showed that the OSI had good construct validity, with *χ*^2^/df = 5.972, RMSEA = 0.043, NFI = 0.987, GFI = 0.989, TLI = 0.977, CFI = 0.989. In this study, Cronbach’s alpha coefficient for the scale was 0.777.

#### 2.2.4 Self-control

The Self-Control Scale (SCS) was used to measure vocational college students’ Self-Control (Tangney et al., 2004). This scale contains 13 items and two subscales, namely, general Self-Discipline (9 items, e.g., “I can effectively handle challenges and stress.”), and impulse control (4 items, e.g., “I possess a well-defined comprehension of my goals and values, and I am diligent in my efforts to achieve them.”). Each item was scored on a 5-point Likert scale (*1* = *strongly disagree*, *5* = *strongly agree*). Responses across theses 13 items were averaged, with higher scores indicating greater self-control. The second-order CFA model showed that the OSI had good construct validity, with *χ*^2^/df = 11.135, RMSEA = 0.061, GFI = 0.976, NFI = 0.987, TLI = 0.975, CFI = 0.988. In this study, the Cronbach’s alpha coefficient for the scale was 0.972.

### 2.3 Procedure

This study received approval from the Ethics Committee of the authors’ university. Prior to data collection, written informed consent was obtained from all participants. In September 2024, participants completed questionnaires assessing boredom proneness, academic disengagement, academic self-efficacy, and self-control via a widely utilized online survey platform (www.wjx.cn). They were assured of the survey’s anonymity and confidentiality and were free to withdraw from participation at any time.

### 2.4 Data analysis

First, the present study calculated descriptive statistics for the variables of interest, followed by Pearson’s correlation analysis among these variables (see Table 1). Second, PROCESS macro (Model 4) developed by Hayes (2013) was used to test the mediating effect of academic self-efficacy in the relationship between boredom proneness and academic disengagement (see Table 2). Third, PROCESS macro (Model 58) was used to test whether self-control moderated the indirect relationship between boredom proneness and academic disengagement through academic self-efficacy (see Table 3). The bootstrapping method based on 5,000 resamples was used to examine the significance of the indirect and conditional indirect effects.

**Table 1:** Correlational analyses for the study variables.

Variables	*M*	*D*	1	2	3	4
Boredom proneness	4.619	0.817	1			
Academic self-efficacy	2.592	0.484	−0.612**	1		
Academic disengagement	3.642	0.599	0.292**	−0.277**	1	
Self-control	2.439	0.752	−0.293**	0.266**	−0.463**	1

*p < 0.05, **p < 0.01, ***p < 0.001.

**Table 2:** Testing the mediation effect of boredom proneness on academic disengagement.

Variables	Model 1 (academic disengagement)				Model 2 (academic self-efficacy)				Model 3(academic disengagement)			
	*β*	*SE*	*LLCI*	*ULCI*	*β*	*SE*	*LLCI*	*ULCI*	*β*	*SE*	*LLCI*	*ULCI*
Boredom proneness	0.291	0.018	0.266	0.338	−0.612	0.015	−0.642	−0.582	0.194	0.023	0.149	0.240
Academic self-efficacy									−0.158	0.023	−0.203	−0.113
*R* ^2^	0.085				0.375				0.101			
*F*	249.619^***^				1611.983^***^				150.269^***^			

*β* are standardized coefficients. SE, standard error; LLCI, lower limit of the 95% confidence interval, ULCI, upper limit of the 95% confidence interval. ***p < 0.001.

**Table 3:** Testing the moderated mediation effect of boredom proneness on academic disengagement.

Variables	Model 1 (academic self-efficacy)				Model 2 (academic disengagement)			
	*β*	*SE*	*LLCI*	*ULCI*	*β*	*SE*	*LLCI*	*ULCI*
Boredom proneness	−0.587	0.016	−0.618	−0.556				
Self-control	0.105	0.017	0.072	0.138				
Self-control × boredom proneness	−0.023	0.012	−0.046	−0.001				
Academic self-efficacy					−0.119	0.022	−0.162	−0.077
Self-control					−0.384	0.019	−0.418	−0.345
Academic self-efficacy × self control					−0.076	0.012	−0.101	−0.053
*R* ^2^	0.384				0.220			
*F*	558.193***				189.510***			

*β* are standardized coefficients. SE, standard error; LLCI, lower limit of the 95% confidence interval; ULCI, upper limit of the 95% confidence interval. Each column is a regression model that predicts the criterion at the top of the column. *p < 0.05, **p < 0.01, ***p < 0.001.

## 3 Results

### 3.1 Bivariate correlation analysis

Results of bivariate correlation analysis for the variables of interest were shown in Table 1. Boredom proneness is positively associated with academic disengagement (*r* = 0.292, *p* < 0.01), and inversely related to academic self-efficacy (*r* = −0.612, *p* < 0.01) and self-control (*r* = −0.293, *p* < 0.01). There is a negative correlation between academic self-efficacy and academic disengagement (*r* = −0.2777, *p* < 0.01), and a positive correlation with self-control (*r* = 0.266, *p* < 0.01). Additionally, academic disengagement exhibits a negative correlation with self-control (*r* = −0.463, *p* < 0.01). Consequently, hypothesis 1 hypothesis 1 was supported.

### 3.2 Mediating role of academic self-efficacy

We used Model 4 of the PROCESS macro (Hayes, 2013) to examine whether academic self-efficacy mediated the relationship between boredom proneness and academic disengagement. As shown in Table 2, boredom proneness negatively predicted academic self-efficacy (*β* = −0.612, *p* < 0.001), and academic self-efficacy negatively predicted academic disengagement (*β* = −0.158, *p* < 0.001). The bias-corrected percentile bootstrap analysis based on 5,000 samples showed that the indirect effect of boredom proneness on academic disengagement through academic self-efficacy was significant (indirect effect = 0.097, *SE* = 0.017, 95% CI = [0.066, 0.131]). The direct effect of boredom proneness on academic disengagement also remained significant (direct effect = 0.195, *SE* = 0.023, 95% *CI* = [0.149, 0.240]). Thus, academic self-efficacy partially mediated the relationship between boredom proneness and academic disengagement, accounting for 33.26% of the total effect.

### 3.3 Moderation effect of self-control

We used Model 58 of the PROCESS macro (Hayes, 2013) to examine whether self-control moderated the first and second stages of the indirect relationship between boredom proneness and academic disengagement through academic self-efficacy.

As shown in Table 3, boredom proneness negatively predicted academic self-efficacy (*β* = −0.587, *p* < 0.001). The interaction between boredom proneness and self-control was significant (*β* = −0.023, *p* < 0.05), indicating that self-control moderated the relationship between boredom proneness and academic self-efficacy. Simple slope analyses showed that boredom proneness was negatively associated with academic self-efficacy at both high (*β*_simple_ = −0.610, *p* < 0.001) and low levels of self-control (*β*_simple_ = −0.564, *p* < 0.001). The negative association was slightly stronger among students with high self-control.

Academic self-efficacy negatively predicted academic disengagement (*β* = −0.119, *p* < 0.001). The interaction between academic self-efficacy and self-control was also significant (*β* = −0.076, *p* < 0.01), indicating that self-control moderated the relationship between academic self-efficacy and academic disengagement. Simple slope analyses showed that the negative relationship was weaker among students with low self-control (*β*_simple_ = −0.035, *p* < 0.001) and stronger among students with high self-control (*β*_simple_ = −0.189, *p* < 0.001).

Bias-corrected percentile bootstrap analyses further showed that the conditional indirect effect of boredom proneness on academic disengagement through academic self-efficacy was not statistically significant at a low level of self-control (indirect effect = 0.023, *SE* = 0.022, 95% *CI* = [−0.017, 0.070]), because the confidence interval included zero. In contrast, the conditional indirect effect was significant at a high level of self-control (indirect effect = 0.119, *SE* = 0.020, 95% *CI* = [0.081, 0.161]).

In addition, as shown in Table 3, academic self-efficacy was negatively correlated with academic disengagement (*β* = −0.119, *p* < 0.001). The interaction between academic self-efficacy and academic disengagement (*β* = −0.076, *p* < 0.01) (see Model 2). In other words, self-control moderated the relationship between academic self-efficacy and academic disengagement. As shown in Figure 3, the relationship academic self-efficacy and academic disengagement at low self-control (*M* – 1*SD*) and at high self-control (*M* + 1*SD*) was plotted. Simple slope tests showed that for students with low self-control, the relationship between academic self-efficacy and academic disengagement was weaker (*β*_simple_ = −0.035, *p* < 0.001); while for students with high low self-control, the relationship between academic self-efficacy and academic disengagement was stronger (*β*_simple_ = −0 0.189, *p* < 0.001).

**Figure 2:**
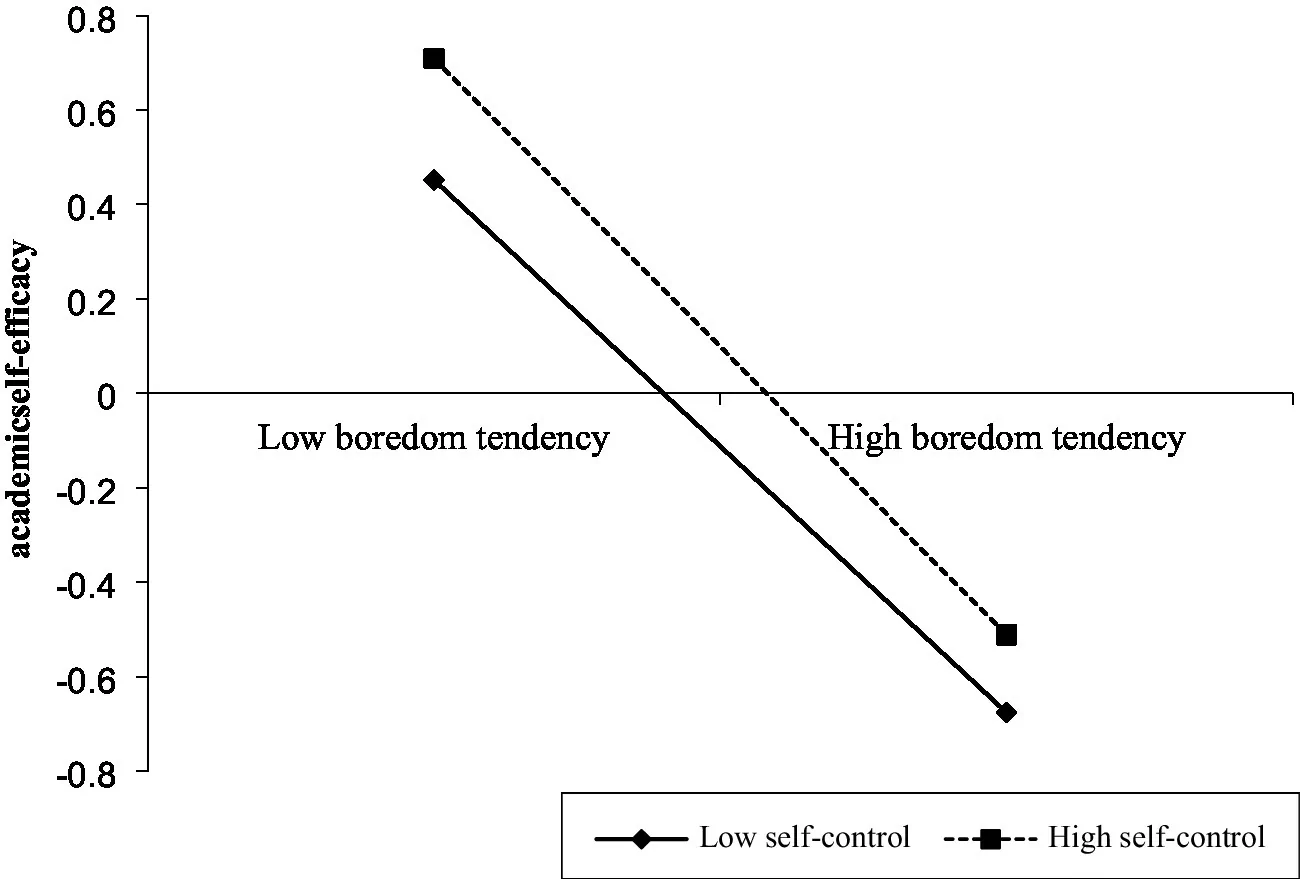
Self-control moderates the relationship between boredom proneness and academic self-efficacy.

**Figure 3:**
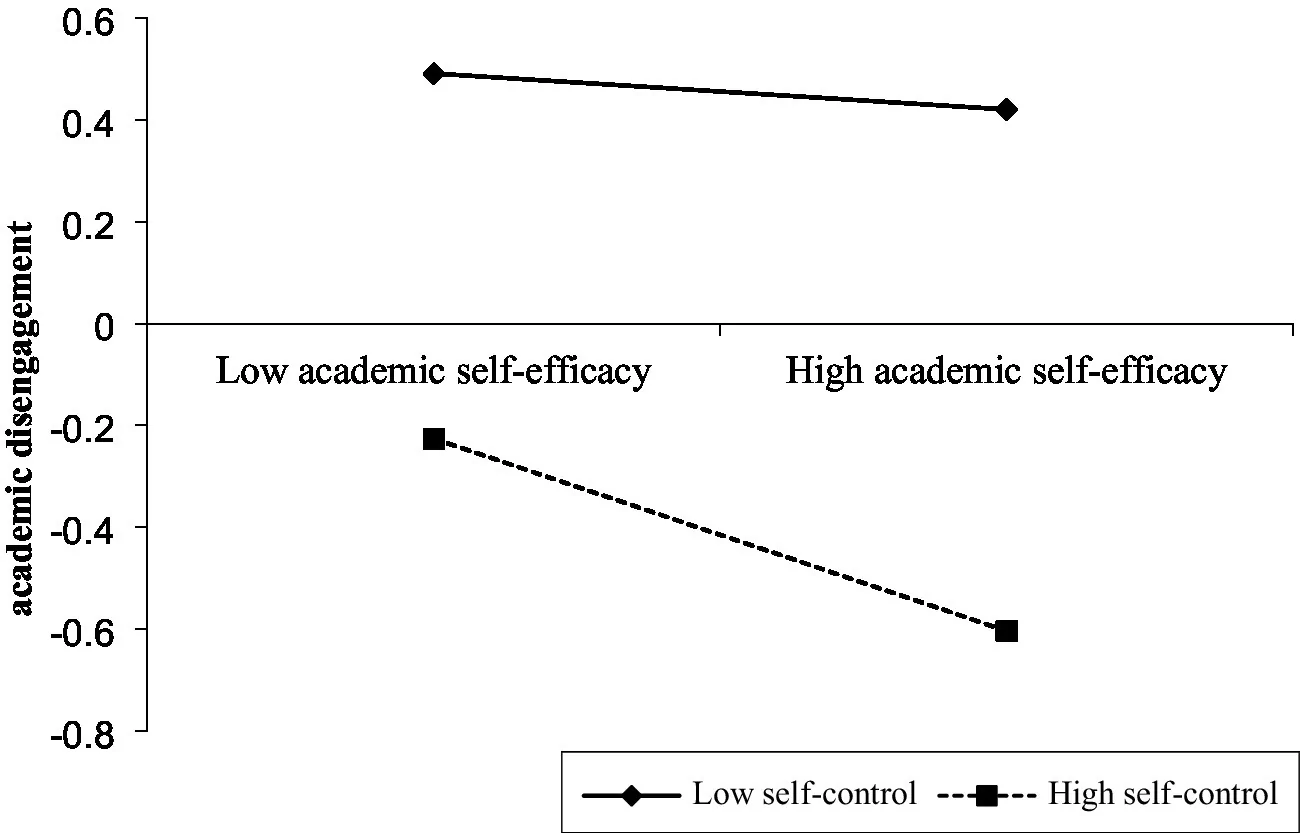
Self-control moderates the relationship between academic self-efficacy and academic disengagement.

Finally, the bias-corrected percentile bootstrap analyses examined the indirect effect of boredom proneness on academic disengagement via academic self-efficacy was moderated self-control. Results showed that for students with low self-control, the indirect relationship between boredom proneness and academic disengagement was weaker (*β* = 0.023, SE = 0.022, 95%CI = [−0.017, 0.070]). While for students with high self-control, the indirect relationship between boredom proneness and academic disengagement was stronger (*β* = 0 0.119, SE = 0 0.020, 95%CI = [0.081, 0.161]).

## 4 Discussion

Although empirical studies have identified the significant impact of boredom proneness on academic disengagement among vocational college students (Ding and Si, 2024; Zhang, 2024), there has been limited research on the psychological mechanisms through which boredom proneness influences academic disengagement. This study, taking vocational college students as participants, explored the mediating role of academic self-efficacy in the relationship between boredom proneness and academic disengagement, as well as the moderating role of self-control in this relationship. Pearson correlation results revealed a significant positive correlation between boredom proneness and academic disengagement. The mediation analysis showed that academic self-efficacy partially mediated the relationship between boredom proneness and academic disengagement. The moderation analysis indicated that self-control significantly moderated both the direct and indirect relationships between boredom proneness and academic disengagement. Specifically, simple slope analyses showed that the negative relationship between boredom proneness and academic self-efficacy was slightly stronger under high self-control than under low self-control. The relationship between academic self-efficacy and academic disengagement was also stronger under high self-control. The conditional indirect effect was significant at high self-control, whereas it was weaker and non-significant at low self-control. These findings contribute to a deeper understanding of the psychological mechanisms by which boredom proneness influences academic disengagement among vocational college students under the condition of self-control.

### 4.1 The mediating role of academic self-efficacy

The results showed that there was a significant negative correlation between boredom proneness and academic efficacy, and there was a significant negative correlation between academic efficacy and weariness behavior of vocational college students. In other words, academic efficacy plays a mediating role in the relationship between boredom and weariness. The results not only support the hypothesis that academic self-efficacy mediates the academic disengagement behavior, but also reveal the mechanism that boredom affects academic disengagement behavior indirectly through academic self-efficacy. Specifically, the boredom proneness is directly related to the students’ weariness, and indirectly through the role of academic efficacy.

To further explore the mediating role of academic efficacy between boredom and weariness, we analyze two indirect paths. First, the study found that boredom proneness and vocational college students’ academic efficacy is significantly negative correlation, which indicates that higher boredom proneness is usually accompanied by lower academic efficacy. This result is consistent with existing studies (Jaradat, 2018; Arafa and Jondi, 2024; Chen, 2025). According to the theory of cognitive load (Sweller and Chandler, 1991) learning effect depends on the effective allocation and use of cognitive resources. When student are bored, negative emotion (such as anxiety and fatigue) occupy part of their cognitive resources, cause distraction and difficulty in concentrating on learning task, lead to a decrease in attention to learning content and a decrease in learning investment (Macklem, 2015; Chen, 2025). In addition, due to excessive consumption of cognitive resources, students may adopt inefficient learning efficacy (surface learning or avoidance learning strategies), reduce academic achievement and affect academic efficacy (Hu and Yeo, 2020; Brockbank and Feldon, 2024).

Secondly, the significant negative correlation between academic efficacy and students’ weariness further verifies the important role of academic efficacy in academic disengagement. The motivation-emotion regulation model shows that students’ academic efficacy not only affects their learning motivation, but also affects their emotion regulation ability (Black and Allen, 2018; Gunzenhauser et al., 2013; Webster and Hadwin, 2015). Students with high academic efficacy have strong emotion regulation ability. When they encounter academic difficulties, they can maintain positive emotional state, overcome negative emotional influences such as anxiety, boredom and pressure, and maintain high academic engagement. On the contrary, students with low academic efficacy lack confidence, are prone to negative emotions such as boredom, anxiety and self-doubt, and are more difficult to manage their emotions when encountering academic difficulties and challenges, which may lead to academic burnout and avoidance behavior, further exacerbating academic disengagement (Rahmati, 2015; Liu and Lu, 2014).

In conclusion, the results not only provide theoretical support for the influence of boredom on academic behavior, but also provide specific guidance for educational practice. The future educational intervention should focus on improving students academic efficacy, so as to help students cope with negative emotions better and reduce the phenomenon of academic disengagement. Vocational schools, for example, should provide personalized coaching to help students set reasonable goals, develop successful experiences, and offer courses in emotion regulation and stress management. In addition, diversified and interactive teaching methods, such as project-based learning and case discussion, are adopted to enhance the relevance of course content and professional needs and enhance students’ learning motivation.

### 4.2 Moderating effect of self-control

Self-control plays an important moderating role between boredom proneness and academic self-efficacy. Emotion regulation theory and stress-resource models suggest that self-control maintains academic self-efficacy by regulating boredom and acts as an emotion regulation resource to relieve learning stress and enhance learning motivation and engagement (Lu and Rameli, 2023; Fritea and Fritea, 2013). Although previous studies suggest that self-control is closely related to emotion regulation and boredom management (Dai, 2023; Struk et al., 2016; Kokkonen and Pulkkinen, 1999; Zhang et al., 2023; Affandi et al., 2025), the present results do not support a simple buffering interpretation of self-control. The negative association between boredom proneness and academic self-efficacy was slightly stronger under high self-control than under low self-control. Thus, self-control should not be interpreted as uniformly mitigating the negative effect of boredom proneness in this model.

Self-control also moderates the relationship between academic self-efficacy and academic disengagement. Cognitive Behavior Theory (CBT) and Self-regulation Theory hold that individual emotions and behaviors are influenced by cognitive assessment, and self-control plays a key role in emotional regulation in academic situations. Studies have shown that individuals with high self-control are able to effectively manage negative emotions (such as anxiety, boredom, and frustration) through self-monitoring, emotion regulation and behavioral adjustment mechanisms (Li et al., 2024), reducing the negative impact of these emotions on academic task performance (Putwain et al., 2013; King and Gaerlan, 2014), maintain high academic self-efficacy and reduce the occurrence of academic avoidance behavior. Conversely, individuals with low self-control, due to lack of effective regulation (Stiller, 2020), are more likely to be dominated by negative emotions, leading to negative cognitive assessments of academic tasks, thereby weakening academic self-efficacy and increasing avoidance behaviors (Villavicencio and Bernardo, 2013; Stiller, 2020; Bondarenko, 2017). These results support Shin and Kemps (2020) by showing that self-control moderated the association between academic self-efficacy and academic disengagement. However, the moderation pattern should not be interpreted as evidence that self-control uniformly weakens the risk pathway. The negative association between academic self-efficacy and academic disengagement was stronger under high self-control than under low self-control. Future research should test when self-control functions as a protective factor and when it functions as a sensitizing factor in academic motivation, emotion regulation, and academic participation. Educational interventions should combine self-control training with academic self-efficacy support, emotion regulation training, self-monitoring and reflection, academic time management, social support, and cooperative learning.

## 5 Implication

Because this study used a cross-sectional design, the implications should be interpreted as association-based practical suggestions rather than evidence of causal intervention effects. First of all, enhance academic effectiveness. This study suggests that academic efficacy mediates the relationship between boredom and academic disengagement. Therefore, educational intervention should focus on enhancing students academic efficacy. Especially in vocational education, students often face greater academic pressure and lack of self-confidence. Through individualized counseling, reasonable goal setting and successful experience accumulation, students self-confidence can be enhanced, and their academic efficacy can be improved. Secondly, emotional regulation and self-control training should be combined with academic self-efficacy support. Because high self-control strengthened rather than weakened the conditional indirect effect in this study, self-control training should not be presented as a standalone buffer. It should be paired with strategies that help students maintain adaptive efficacy beliefs when boredom occurs. Thirdly, diversification of educational methods. In higher vocational education, traditional teaching methods may fail to stimulate students’ interest in learning, which leads to boredom. Self-determination theory (Ryan and Deci, 2000) emphasizes that learning motivation can be effectively enhanced when individuals satisfy the conditions of autonomy, competence and belonging. The school adopts diversified and interactive teaching methods to enhance students motivation, such as project-based learning and case discussion, so as to enhance students sense of participation in the curriculum, enhance learning motivation and improve academic effectiveness. Finally, under the background of vocational education in China, students’ family background, social expectation and cultural identity have profound influence on their learning behavior. Cultural psychology (Vygotsky, 1978) emphasizes the influence of cultural background on individual development. Education intervention should take cultural factors into full consideration. Home-school cooperation is very important to students academic efficacy. Schools should strengthen communication and cooperation with parents, pay attention to students’ emotional management and academic development together, enhance students’ academic efficacy and reduce academic disengagement.

## 6 Limitations and future directions

Several limitations of this study merit deeper exploration. Firstly, convenience sampling is adopted. The sample was drawn primarily from a single vocational college in Guangxi, which may introduce regional bias and limit the generalizability of findings regarding academic disengagement among vocational college students across China. Future research could expand the sample to cover different provinces and types of vocational colleges. Moreover, the use of multistage sampling or random sampling of students from multiple institutions can further ensure the representativeness of the sample. Secondly, the cross-sectional design of this study limits the inference of causal relationship, especially the effect of boredom proneness on study-weariness behavior under the influence of self-control. Future research should adopt longitudinal design to better explore the dynamic relationship between boredom proneness and self-control in higher vocational college students’ academic disengagement behavior. Third, this study did not control for external factors that may be associated with academic disengagement, such as academic stress and social support. Previous research has suggested that factors (e.g., academic stress) may influence the moderating role of self-control and boredom tendencies in influencing students’ academic behavior (Kula, 2022; Parthi and Gupta, 2017). Future studies should consider controlling for these external factors to better understand the impact of boredom tendencies under self-control on academic disengagement.

Although this study aims to explore the mediating role of academic self-efficacy between boredom proneness and academic disengagement, it provides a new theoretical perspective for vocational college students’ academic behavior. Future studies could further validate other variables that may influence academic disengagement, such as emotional regulation and social support, into the model, and explore how these factors interact with boredom tendencies to influence academic disengagement behavior.

## Data Availability

The original contributions presented in the study are included in the article/supplementary material, further inquiries can be directed to the corresponding author.
