# Correction: Boredom proneness and Chinese vocational college students academic disengagement: mediating role of academic self-efficacy and moderating role of self-control

**DOI:** 10.3389/fpsyg.2026.1794300

**Published:** 2026-03-02

**Authors:** Yantao Shi, Xueli Hui, Guanghai Li, Mingkun Ouyang, Cheng Yang

**Affiliations:** 1 Faculty of Education, Guangxi Normal University, Guilin, China; 2 College of Innovation and Entrepreneurship, Guangxi Minzu University, Nanning, China; 3 College of Preschool Education, Chongqing Youth Vocational & Technical College, Chongqing, China; 4 School of Educational Sciences, Guangxi Minzu University, Nanning, China; 5 School of Foreign Studies, Guangxi Minzu University, Nanning, China

**Keywords:** academic disengagement, academic self-efficacy, boredom proneness, self-control, vocational college students

Affiliation College of Preschool Education, Chongqing Youth Vocational & Technical College, Chongqing, China was erroneously given as Chongqing Youth Vocational and Technical College, Chongqing, China.

Authors Mingkun Ouyang and Cheng Yang were erroneously assigned to affiliation College of Innovation and Entrepreneurship, Guangxi Minzu University, Nanning, China. This affiliation has now been removed for authors Mingkun Ouyang and Cheng Yang.

Affiliation School of Educational Sciences, Guangxi Minzu University, Nanning, China was omitted from the paper. This affiliation has now been added for author Mingkun Ouyang.

Affiliation School of Foreign Studies, Guangxi Minzu University, Nanning, China was omitted from the paper. This affiliation has now been added for author Cheng Yang.

The original version of this article has been updated.

