# Correction: Boredom proneness and Chinese vocational college students academic disengagement: mediating role of academic self-efficacy and moderating role of self-control

**DOI:** 10.3389/fpsyg.2026.1924195

**Published:** 2026-08-12

**Authors:** Yantao Shi, Xueli Hui, Guanghai Li, Mingkun Ouyang, Cheng Yang

**Affiliations:** 1 Faculty of Education, Guangxi Normal University, Guilin, China; 2 College of Innovation and Entrepreneurship, Guangxi Minzu University, Nanning, China; 3 College of Preschool Education, Chongqing Youth Vocational & Technical College, Chongqing, China; 4 School of Educational Sciences, Guangxi Minzu University, Nanning, China; 5 School of Foreign Studies, Guangxi Minzu University, Nanning, China

**Keywords:** academic disengagement, academic self-efficacy, boredom proneness, self-control, vocational college students

A correction has been made to the section **Abstract**, *Method*. The incorrect sentence was written as, “Structural equation modeling (SEM) was used to analyze the data, and moderated mediation analyses were conducted to explore the moderating effects of self-control.” The correct sentence should read as, “Confirmatory factor analysis (CFA), Pearson correlation analyses, and PROCESS macro analyses were used to examine measurement validity and test the mediation and moderated mediation models.”

A correction has been made to the section **Abstract**, *Results*. The incorrect paragraph was written as “The results indicate that boredom proneness positively predicts academic disengagement. Academic self-efficacy was found to partially mediate this association. Subsequently, moderated-mediation analyses further revealed that trait self-control qualifies both the direct and indirect links between boredom proneness and disengagement. When self-control is high, the path from boredom proneness to diminished academic self-efficacy is attenuated and avoidance behaviors remain low; conversely, when self-control is low, the same path is intensified and avoidance behaviors escalate.” The correct paragraph should read as “Boredom proneness positively predicted academic disengagement. Academic self-efficacy partially mediated this association. Self-control moderated the path from boredom proneness to academic self-efficacy and the path from academic self-efficacy to academic disengagement. The indirect effect of boredom proneness on academic disengagement through academic self-efficacy was stronger under high self-control, whereas it was weaker and non-significant under low self-control.”

A correction has been made to the section **Abstract**, *Conclusion*. The incorrect paragraph was written as “This study elucidates the mechanisms connecting boredom proneness to academic disengagement in vocational-college students by demonstrating that self-control significantly moderates this pathway. The findings imply that interventions designed to strengthen self-control may attenuate academic disengagement within this population.” The correct paragraph should read as “Self-control should not be interpreted as a simple buffering factor in this model. Interventions should combine academic self-efficacy support with self-regulatory support.”

A correction has been made to section **1 Introduction**, sub-section *1.1 Boredom proneness and academic disengagement*, first sentence. The incorrect sentence was written as, “Boredom proneness has a significant negative impact on individuals' learning attitudes and behaviors, with a negative correlation between boredom proneness and academic disengagement.” The correct sentence should read as, “Boredom proneness is positively associated with academic disengagement.”

A correction has been made to section **1 Introduction**, sub-section *1.3 Moderating role of self-control*, paragraph 2. The incorrect sentence was written as, “Secondly, we propose that self-control plays a mediating role between academic efficacy and academic disengagement.” The correct sentence should read as, “Secondly, self-control is proposed as a moderator of the relationship between academic self-efficacy and academic disengagement.”

A correction has been made to section **2 Methods**, sub-section *2.2.2 Academic disengagement*. The incorrect sentence was written as, “Each item was rated on a 5-point Likert scale from ranging from 1 (completely consistent) to 5 (completely inconsistent), and higher average scores indicated higher levels of academic disengagement.” The correct sentence should read as, “Each item was rated on a 5-point Likert scale from 1 (completely inconsistent) to 5 (completely consistent), and higher average scores indicated higher levels of academic disengagement.”

A correction has been made to section **2 Methods**, sub-section *2.2.3 Academic self-efficacy*, second sentence. The incorrect sentence was written as, “The scale consists of 11 items (e.g., “I have found that I tend to daydream during class, which prevents me from focusing on the lecture.”).” The correct sentence should read as, “The scale consists of 11 items (e.g., “When thinking about a problem, I can connect the knowledge I have learned before and after.”).”

A correction has been made to section **2 Methods**, sub-section *2.4 Data analysis*, third sentence. The incorrect sentence was written as, “Third, PROCESS macro (Model 58) was used to test the moderating effect of psychological resilience in the direct and indirect relationships between boredom proneness and academic disengageen (see Table 3). The bootsrapping method based on 5,000 resample was used to examine the significance of the direct and indirect effects.” The correct sentence should read as, “Third, PROCESS macro (Model 58) was used to test whether self-control moderated the indirect relationship between boredom proneness and academic disengagement through academic self-efficacy (see Table 3). The bootstrapping method based on 5,000 resamples was used to examine the significance of the indirect and conditional indirect effects.”

A correction has been made to section **3 Results**, sub-section *3.2 Mediating role of academic self-efficacy*, last sentence. The incorrect sentence was written as, “Thus, self-control acts as a partial mediating role in the relationship between boredom proneness and academic disengagement, which accounted for 33.26% of the total effect.” The correct sentence should read as, “Thus, academic self-efficacy acts as a partial mediator in the relationship between boredom proneness and academic disengagement, accounting for 33.26% of the total effect.”

A correction has been made to section **3 Results**, sub-section *3.3 Moderation effect of self-control*, paragraph two. The incorrect paragraph was written as, “In other words, self-control moderated the relationship between boredom proneness and academic disengagement. The interaction between academic self-efficacy and academic disengagement (β = −0.076, p < 0.01) (see Model 2). While for students with high low self-control, the relationship between academic self-efficacy and academic disengagement was stronger (βsimple = −0 0.189, p < 0.001). Finally, the bias-corrected percentile bootstrap analyses examined the indirect effect of boredom proneness on academic disengagement via academic self-efficacy was moderated self-control. While for students with high self-control, the indirect relationship between boredom proneness and academic disengagement was stronger (β = 0 0.119, SE = 0 0.020, 95%CI = [0.081, 0.161]).” The correct paragraph should read as, “The results showed a moderated mediation pattern involving both model paths. In Model 2, the interaction between academic self-efficacy and self-control was significant (β = −0.076, *p* < 0.01). For students with high self-control, the relationship between academic self-efficacy and academic disengagement was stronger (βsimple = −0.189, *p* < 0.001). Bias-corrected percentile bootstrap analyses further showed that the indirect effect of boredom proneness on academic disengagement through academic self-efficacy was conditional on self-control. For students with high self-control, the indirect effect was stronger [β = 0.119, SE = 0.020, 95% CI = [0.081, 0.161]].”

A correction has been made to section **4 Discussion**. The incorrect sentence was written as, “Specifically, under high self-control conditions, boredom proneness had a minimal impact on academic self-efficacy; whereas under low self-control conditions, boredom proneness led to a significant decline in academic self-efficacy. Individuals with high self-control typically exhibit higher academic self-efficacy and show lower levels of academic avoidance behavior. In contrast, individuals with low self-control tend to have lower academic self-efficacy and often display higher levels of academic avoidance behavior.” The correct sentence should read as, “Specifically, simple slope analyses showed that the negative relationship between boredom proneness and academic self-efficacy was slightly stronger under high self-control than under low self-control. The relationship between academic self-efficacy and academic disengagement was also stronger under high self-control. The conditional indirect effect was significant at high self-control, whereas it was weaker and non-significant at low self-control.”

A correction has been made to section **4 Discussion**, sub-section *4.2 Moderating effect of self-control*, paragraph one. The incorrect paragraph was written as, “Individuals with high self-control are able to effectively employ emotion-regulating strategies (e.g., through reassessment and attention shifting) to alleviate boredom (Dai, 2023; Struk et al., 2016), which translates these emotions into opportunities for motivation and goal adjustment to maintain high academic self-efficacy. Compared with high self-control individuals, low self-control individuals are more likely to be negatively affected by boredom due to lack of effective emotional regulation resources (Kokkonen and Pulkkinen, 1999), resulting in a significant decline in academic self-efficacy. The results further support existing studies (Zhang et al., 2023; Affandi et al., 2025) and provides a new perspective on educational practice. This study emphasizes the importance of improving students' emotional regulation and self-control ability, especially when students face great academic challenges. This strategy provides effective guarantee for enhancing academic self-efficacy.” The correct paragraph should read as, “Although previous studies suggest that self-control is closely related to emotion regulation and boredom management (Dai, 2023; Struk et al., 2016; Kokkonen and Pulkkinen, 1999; Zhang et al., 2023; Affandi et al., 2025), the present results do not support a simple buffering interpretation of self-control. The negative association between boredom proneness and academic self-efficacy was slightly stronger under high self-control than under low self-control. Thus, self-control should not be interpreted as uniformly mitigating the negative effect of boredom proneness in this model.”

A correction has been made to section **4 Discussion**, sub-section *4.2 Moderating effect of self-control*, paragraph two. The incorrect paragraph was written as, “This study supports, Shin and Kemps (2020) and further reveals the moderating effect of self-control on the relationship between academic self-efficacy and academic disengagement behavior. These results suggest that self-control is not only a key factor in individual emotion regulation, but also plays an important role in predicting academic behavior. Therefore, future research will focus on exploring the mechanism of self-control in academic motivation, emotional regulation and academic participation, and combine specific educational interventions (such as emotional regulation training, self-monitoring and reflection, academic time management and social support, cooperative learning, etc.) to help students improve self-control and reduce the frequency of academic disengagement.” The correct paragraph should read as, “These results support Shin and Kemps (2020) by showing that self-control moderated the association between academic self-efficacy and academic disengagement. However, the moderation pattern should not be interpreted as evidence that self-control uniformly weakens the risk pathway. The negative association between academic self-efficacy and academic disengagement was stronger under high self-control than under low self-control. Future research should test when self-control functions as a protective factor and when it functions as a sensitizing factor in academic motivation, emotion regulation, and academic participation. Educational interventions should combine self-control training with academic self-efficacy support, emotion regulation training, self-monitoring and reflection, academic time management, social support, and cooperative learning.”

A correction has been made to section **5 Implication**, first sentence. The incorrect sentence was written as, “This study reveals the complex psychological mechanism that influences vocational college students academic behavior by verifying the mediating role of academic self-efficacy between boredom proneness and academic disengagement, and the moderating role of self-control in two paths.” The correct sentence should read as, “Because this study used a cross-sectional design, the implications should be interpreted as association-based practical suggestions rather than evidence of causal intervention effects.”

A correction has been made to section **5 Implication**, paragraph one. The incorrect paragraph was written as, “Secondly, the cultivation of emotional regulation and self-control ability. Studies have shown that self-control plays a key role in emotional regulation, especially in the face of academic challenges (Baumeister et al., 2007). Students in vocational education are often affected by academic pressure and negative emotions. Schools can offer emotional regulation and stress management courses to improve students' emotional regulation skills, help them cope with academic difficulties, maintain positive emotions and reduce the risk of academic disengagement.” The paragraph sentence should read as, “Secondly, emotional regulation and self-control training should be combined with academic self-efficacy support. Because high self-control strengthened rather than weakened the conditional indirect effect in this study, self-control training should not be presented as a standalone buffer. It should be paired with strategies that help students maintain adaptive efficacy beliefs when boredom occurs.”

There was a mistake in Table 3 as published. In the F row, the values were incorrectly displayed as “558.193^**^” and “189510^**^”. The corrected Table 3 appears below.

**Table 3:** Testing the moderated mediation effect of boredom proneness on academic disengagement.

Variables	Model 1 (academic self-efficacy)				Model 2 (academic disengagement)			
	β	SE	LLCI	ULCI	β	SE	LLCI	ULCI
Boredom proneness	−0.587	0.016	−0.618	−0.556				
Self-control	0.105	0.017	0.072	0.138				
Self-control × boredom proneness	−0.023	0.012	−0.046	−0.001				
Academic self-efficacy					−0.119	0.022	−0.162	−0.077
Self-control					−0.384	0.019	−0.418	−0.345
Academic self-efficacy × self-control					−0.076	0.012	−0.101	−0.053
*R* ^2^	0.384				0.220			
*F*	558.193^***^				189.510^***^			

β are standardized coefficients. SE, standard error; LLCI, lower limit of the 95% confidence interval; ULCI, upper limit of the 95% confidence interval. Each column is a regression model that predicts the criterion at the top of the column. ^*^p < 0.05, ^**^p < 0.01, ^***^p < 0.001.

There was a mistake in the caption of Figure 2 as published. The caption incorrectly identified the moderated relationship. The corrected caption of Figure 2 appears below.

“Figure 2. Self-control moderates the relationship between boredom proneness and academic self-efficacy.”

There was a mistake in the caption of Figure 3 as published. The caption incorrectly identified the moderated relationship. The corrected caption of Figure 3 appears below.

“Figure 3. Self-control moderates the relationship between academic self-efficacy and academic disengagement.”

The original version of this article has been updated.

